# Laser-Based Photobiomodulation in Postoperative Tissue Healing in Oral and Maxillofacial Surgery: Systematic Review of RCTs

**DOI:** 10.3390/jcm15020613

**Published:** 2026-01-12

**Authors:** Iwona Niedzielska, Grzegorz Dawiec, Rafał Wiench, Małgorzata Pihut, Dariusz Skaba, Josep Arnabat-Dominguez

**Affiliations:** 1 Department of Cranio-Maxillofacial Surgery, Faculty of Medical Sciences, Medical University of Silesia, 40-027 Katowice, Poland; 2 Department of Periodontal and Oral Mucosa Diseases, Faculty of Medical Sciences in Zabrze, Medical University of Silesia, 40-055 Katowice, Poland; 3 Prosthodontic and Orthodontic Department, Jagiellonian University Medical College, 4 Montelupich Str., 31-155 Krakow, Poland; 4 Faculty of Medicine and Health Sciences, Dental School, University of Barcelona, IDIBELL Institute, 08908 Barcelona, Spain

**Keywords:** photobiomodulation, low-level laser therapy, oral surgery, maxillofacial surgery, bone remodeling, osseointegration, implant stability, soft tissue healing, postoperative recovery, systematic review

## Abstract

**Background**: Postoperative bone healing can be impaired by systemic factors and surgical trauma, leading to delayed recovery. Photobiomodulation therapy (PBMT) has been proposed as a non-invasive method to enhance osteogenesis, but variability in protocols and outcomes limits its clinical use. **Aim**: To systematically review and synthesize evidence from randomized controlled trials (RCTs) evaluating PBMT’s effectiveness in promoting postoperative osteogenesis. **Methods**: A systematic search of PubMed, Embase, Scopus, and Cochrane Library was conducted following the PRISMA 2020 guidelines. Only RCTs comparing PBMT with sham treatment or standard care were included. Data on laser parameters, surgical indications, and outcomes such as bone regeneration, healing time, and implant stability were extracted. The risk of bias of the included randomized studies was evaluated using the Cochrane Risk of Bias 2 (RoB version 2) tool. **Results**: Twelve RCTs were included. PBMT consistently improved early soft tissue healing and reduced postoperative inflammation and edema. Some studies showed accelerated bone maturation, especially in grafted sockets and distraction osteogenesis, while others reported no significant long-term effects on implant stability or chronic lesion healing. Heterogeneity in laser parameters limited comparability. **Conclusions**: PBMT is a safe adjunct that reliably enhances early postoperative healing and may promote bone remodeling in selected cases. Standardized protocols and larger, high-quality RCTs are needed to confirm long-term benefits and optimize treatment parameters.

## 1. Introduction

Postoperative bone healing is a multifaceted physiological process involving tightly regulated cellular, molecular, and vascular events [1,2,3]. While advances in surgical technique, biomaterials, and pharmacological support have improved outcomes in bone regeneration, incomplete or delayed healing continues to present a significant clinical challenge across multiple disciplines, including orthopedic, maxillofacial, and dental surgery. Factors such as patient age, systemic disease, smoking, and surgical trauma can impair osteogenesis, prolong recovery, and compromise functional outcomes [4,5,6]. Consequently, there is an ongoing search for adjunctive therapies that can enhance the body’s natural regenerative capacity without introducing significant risk or complexity. Laser-based biostimulation, commonly referred to as photobiomodulation therapy (PBMT) or previously low-level laser therapy (LLLT), has emerged as one of the most promising non-invasive modalities to address this need. PBMT harnesses the therapeutic potential of specific wavelengths of light to initiate photochemical reactions within target tissues, particularly within the mitochondria of bone-forming cells [7,8,9,10]. Through the precise modulation of cellular energy metabolism, oxidative stress, and growth factor expression, PBMT promotes osteoblast proliferation, accelerates extracellular matrix synthesis, and stimulates angiogenesis, key elements in efficient bone repair. The appeal of PBMT lies not only in its biological efficacy but also in its favorable safety profile and ease of clinical application. By delivering controlled doses of red or near-infrared light directly to surgical sites, clinicians can potentially reduce healing time, minimize postoperative discomfort, and enhance the quality of regenerated bone [11,12,13,14,15]. This therapeutic approach is supported by a growing body of evidence from experimental and clinical studies demonstrating measurable improvements in bone density, structural integrity, and patient recovery timelines. Despite its promise, the widespread adoption of PBMT in postoperative osteogenesis has been limited by variability in treatment parameters, including wavelength, energy density, and application frequency [16,17,18]. Such variability complicates a direct comparison of study outcomes and underscores the need for standardized protocols. As research advances, integrating PBMT into evidence-based oral and maxillofacial surgical care may enhance postoperative recovery and bone management, shifting the focus toward biologically driven, patient-specific regeneration strategies.

This systematic review aims to compile and critically evaluate randomized controlled trials investigating the effects of PBMT on postoperative outcomes, including soft tissue healing, pain, inflammation, and bone-related measures such as bone remodeling and implant stability, to provide a comprehensive understanding of its efficacy, limitations, and future directions for clinical application in dentistry and maxillofacial surgery. While the primary focus is postoperative osteogenesis, we also synthesize evidence on soft tissue healing and early inflammatory control to reflect PBM’s broader clinical effects.

## 2. Materials and Methods

### 2.1. Focused Question

This systematic review was developed using the PICO [19] (Population, Intervention, Comparison, Outcome) framework to define the research question: In patients undergoing surgical procedures requiring bone healing (Population), does postoperative laser-based biostimulation (Intervention) enhance osteogenesis, as measured by bone regeneration, mineralization rate, or healing time (Outcome), compared with standard postoperative care, sham treatment, or no intervention (Comparison)?

### 2.2. Search Strategy

**Figure 1:**
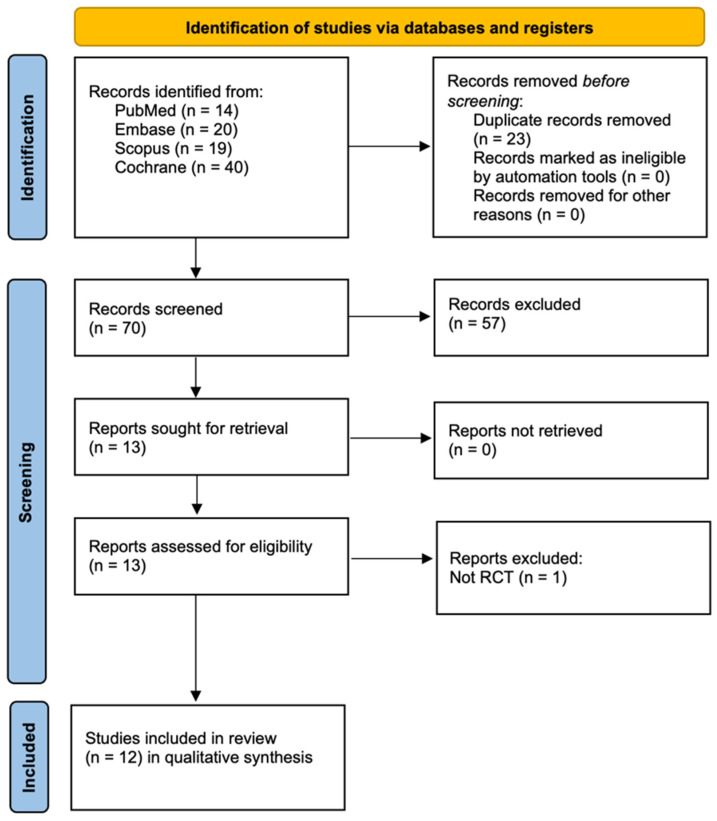
Prisma 2020 flow diagram.

This systematic review, registered in the PROSPERO database [20] (Registration ID: CRD420251151594), was designed and reported in accordance with the PRISMA 2020 guidelines to ensure methodological transparency and scientific rigor (Supplementary Material) [21]. The search was conducted until 20 August 2025. A comprehensive literature search was performed across four major electronic databases: PubMed/MEDLINE, Embase, Scopus, and the Cochrane Library to identify randomized controlled trials investigating the effects of laser-based biostimulation on postoperative osteogenesis. The complete search strategy is provided in Figure 1. Searches were conducted independently by three reviewers using a standardized set of keywords and controlled vocabulary terms tailored to capture studies involving laser therapy applied to promote bone healing following surgical interventions. Only English-language publications were considered, with no restrictions on publication year. The selection process involved an initial screening of titles and abstracts, followed by full-text review conducted independently by two reviewers based on predefined eligibility criteria outlined in Table 1. Any disagreements were resolved through consensus discussion.

### 2.3. Study Selection Process

To ensure methodological rigor and reduce potential bias, all studies identified through the search were screened in a structured, multi-phase process conducted independently by at least two reviewers. The initial screening of titles and abstracts was guided by predefined inclusion criteria, and disagreements were resolved through consensus discussion to maintain consistency and objectivity. Eligible studies were randomized controlled trials evaluating the effects of laser-based biostimulation on postoperative osteogenesis. Inclusion required the assessment of outcomes such as bone regeneration, bone density, mineralization rate, or healing time following surgical interventions. Only trials employing rigorous designs with appropriate control groups (placebo, sham irradiation, or standard postoperative care) were considered. Studies also had to clearly report treatment parameters, patient selection criteria, and follow-up periods sufficient to evaluate the persistence of effects. Exclusion criteria included non-peer-reviewed sources (e.g., conference abstracts, case reports, editorials, opinion pieces, book chapters, unpublished theses), non-English publications, duplicate reports without novel findings, and studies lacking scientific rigor. Research not primarily focused on postoperative osteogenesis or not employing laser-based biostimulation as the main intervention was excluded, as were trials without a control/comparison group or those using unrelated modalities. In vitro or preclinical studies conducted under laboratory conditions without direct clinical applicability were also omitted.

### 2.4. Risk of Bias in Individual Studies

To safeguard methodological integrity and minimize potential selection bias, all identified records underwent independent evaluation by at least two reviewers during the screening process. Agreement between reviewers was quantified using Cohen’s kappa statistic to assess inter-rater reliability. Discrepancies in study inclusion decisions were resolved through structured discussion until consensus was reached. This transparent, standardized approach was applied to ensure that only randomized controlled trials meeting the eligibility criteria for evaluating laser-based biostimulation in postoperative osteogenesis were included. The process was designed to strengthen the reliability of the review and promote an unbiased assessment of the available evidence.

### 2.5. Quality Assessment

The risk of bias of the included randomized studies was evaluated using the Cochrane Risk of Bias 2 (RoB 2) tool. This tool assesses potential bias across five domains: the randomization process, deviations from intended interventions, missing outcome data, measurement of the outcome, and selection of the reported result. Each domain was judged as low risk or as having some concerns, leading to an overall risk of bias judgment for each study. The assessment was performed systematically to ensure a consistent and transparent appraisal of methodological quality [22].

The certainty of the evidence was graded using the Grading of Recommendations Assessment, Development and Evaluation (GRADE) approach [23]. Evidence was initially rated according to study design and subsequently assessed across five domains: risk of bias, inconsistency, indirectness, imprecision, and publication bias. The overall certainty of evidence for each outcome was classified as high, moderate, low, or very low. This process was applied systematically to provide a transparent and structured evaluation of the strength and reliability of the evidence supporting the study findings.

### 2.6. Data Extraction

Two reviewers independently recorded for each trial: bibliographic details, country, clinical setting, design, sample size, eligibility criteria, participant demographics, surgical indication and site, comparator type, and follow-up length. Laser information was captured at high granularity: device type and model, wavelength, power output, beam mode, spot size, dose per point, fluence, exposure time, number and location of application points, delivery route (contact or non-contact, intraoral or extraoral), session frequency, and total number of sessions, plus any co-interventions. Outcomes collected included objective osteogenesis measures (radiographic bone fill, bone mineral density, histomorphometry, ISQ, time to clinical healing), patient-reported pain and edema, functional recovery, adverse events, and attrition.

## 3. Results

### 3.1. Risk of Bias and Evidence Assessment

The risk of bias and overall quality of evidence of the included studies were systematically evaluated to determine the internal validity and reliability of the findings. Assessment was conducted across five predefined domains: randomization process, deviations from intended interventions, missing outcome data, outcome measurement, and selective reporting. Each study was judged for each domain and subsequently assigned an overall risk of bias rating. The results of this assessment are summarized in Table 2, providing a transparent overview of the methodological strengths and potential limitations across the included studies. The studies rated as having some concerns primarily showed limitations in specific methodological domains rather than across all aspects of study design. 

Concerns related to the randomization process (D1) were observed in García-Morales et al. (2011) [29], where insufficient detail was provided regarding sequence generation or allocation concealment. Deviations from intended interventions (D2) raised some concerns in Monea et al. (2015) [32] and Özkan et al. (2015) [33], largely due to limited reporting on adherence to intervention protocols or lack of clarity on whether deviations were balanced between groups. Missing outcome data (D3) contributed to some concerns in AboElsaad et al. (2009) [24], suggesting potential attrition or incomplete reporting of outcomes. As a result of these domain-level issues, the overall risk of bias for these studies was judged as having some concerns, while outcome measurement and selective reporting were generally assessed as low risk across the included literature.

The certainty of evidence for each clinically relevant outcome was evaluated using the GRADE approach and is summarized in Table 3. All outcomes were derived from randomized controlled trials and therefore initially rated as high certainty. Judgments were informed by the overall low risk of bias across studies, the direct applicability of the interventions and outcomes to clinical practice, and the absence of serious safety concerns. Where variability in results or differences in laser protocols were observed, the certainty of evidence was rated as moderate rather than downgraded further, reflecting that such heterogeneity was largely clinical and methodological rather than contradictory in effect direction. Overall, the GRADE assessment indicates high to moderate certainty that photobiomodulation therapy provides reliable benefits for early postoperative healing and is a safe adjunctive intervention, while its effects on bone-related outcomes and implant stability remain more context dependent.

In addition to the formal assessment of risk of bias and certainty of evidence, important clinical modifiers of postoperative healing were considered when interpreting the findings of the included trials. Patient-related comorbidities, particularly diabetes mellitus and smoking, are well-established factors that can adversely affect soft tissue repair, angiogenesis, immune response, and bone remodeling after oral and maxillofacial surgical procedures. Diabetes is associated with impaired osteoblast function, reduced collagen synthesis, and delayed neovascularization, while smoking negatively influences tissue oxygenation, inflammatory response, and osseointegration, ultimately increasing the risk of delayed or compromised healing [4,5,6,17,18]. Although some included studies reported general health status or excluded patients with severe systemic disease, most trials did not provide detailed stratification or subgroup analyses based on these comorbidities. As a result, the potential modifying effect of diabetes, smoking status, or other systemic conditions on the response to photobiomodulation therapy could not be formally assessed within the GRADE framework. This limitation was considered during evidence interpretation, as variability in patient risk profiles may partly explain heterogeneity in observed outcomes across studies. Another methodological limitation relevant to evidence grading was the lack of a standardized summary of baseline bone quality and surgical protocols across trials. Bone density, cortical thickness, defect morphology, and local biological environment are critical determinants of postoperative osteogenesis and implant stability, and they strongly influence healing trajectories regardless of adjunctive interventions [1,2,3,10,14]. While some studies specified bone quality classifications, grafting procedures, or anatomical sites, many did not report baseline bone characteristics in a uniform or quantitative manner. Similarly, surgical protocols varied considerably with respect to flap design, graft materials, implant systems, and postoperative care, and these details were inconsistently documented. The absence of standardized reporting of baseline bone conditions and surgical techniques limited direct comparability between studies and reduced the ability to attribute observed effects solely to photobiomodulation therapy. Within the GRADE assessment, this issue was primarily reflected under the domains of inconsistency and indirectness for bone-related outcomes, contributing to moderate rather than high certainty ratings where appropriate.

### 3.2. Study Selection

Figure 1 illustrates the study selection process. A total of 93 records were initially identified from four databases: PubMed (14), Embase (20), Scopus (19), and Cochrane (40). Before screening, 23 duplicate records were removed, leaving 70 records for screening. During the screening phase, 57 records were excluded, and 13 reports were sought for retrieval, all of which were successfully retrieved. These 13 reports were assessed for eligibility, and one was excluded, as it was not an RCT [36]. Ultimately, 12 studies were included in the qualitative synthesis for the review.

### 3.3. Data Presentation

Table 4, Table 5 and Table 6 provide a summary of the extracted details.

### 3.4. Overview of Study Characteristics

Table 4 summarizes 12 clinical studies investigating the effects of PBMT on bone and soft tissue healing in dentistry. It includes details such as the first author and year, country, and the main aim of each study. The studies cover a range of applications, including dental implant stability, third molar extractions, bone regeneration, periapical lesion healing, and osseointegration. Collectively, they provide insight into how different laser protocols, wavelengths, and adjunctive treatments like platelet-rich fibrin influence postoperative outcomes in oral and maxillofacial surgery.

### 3.5. Main Study Outcomes

AboElsaad et al. [24] studied 20 patients with bilateral infra-bony periodontal defects. The test group received bioactive glass plus GaAlAs laser, while the control received only bioactive glass. At 3 months, the laser group showed better probing depth, attachment level, and bone fill, but by 6 months, differences were not significant, indicating early but temporary benefits [24]. Akkaya et al. [25] randomized 40 premolar sockets into four groups: control, PRF, diode laser, and PRF + diode laser. Bone healing was significantly greater in the combined group (*p* = 0.04), while PRF or laser alone showed non-significant improvement [25]. Bozkaya et al. [26] evaluated 93 implants in 22 patients. The test group received PBM, and the control received sham treatment. Implant stability improved in both groups with no significant differences, indicating that PBM did not enhance early stabilization [26]. Camolesi et al. [27] compared dual-wavelength PBM to sham treatment in 40 implants. PBM significantly improved early healing and reduced inflammation (*p* = 0.030) but did not affect implant stability over 8 weeks [27]. Demirok et al. [28] compared L-PRF to PBMT in third molar extractions. No significant difference was found in pain, healing, or bone density. L-PRF provided slightly better early healing, while PBMT improved trabecular bone quality at 3 months [28]. García-Morales et al. [29] tested LLLT on 30 implants. Both groups showed similar stability trends, with no significant effect of LLLT when the baseline bone quality was high [29]. Ismail et al. [30] compared PBM to sham treatment after root canal therapy in 60 patients. Both groups had significant lesion volume reduction, but no difference between groups after 12 months, indicating no added PBM benefit [30]. Mandić et al. [31] studied 44 implants with LLLT vs. control. Stability was higher with LLLT at week 5 (*p* = 0.030), but osseointegration and success rates were similar [31]. Monea et al. [32] found LLLT halved bone healing time in extraction sockets with grafts, showing abundant new bone at 60 days vs. 120 days for controls [32]. Özkan et al. [33] showed LLLT increased bone mineral density and accelerated bone maturation in adolescents undergoing mandibular distraction osteogenesis (*p* < 0.05) [33]. Mandić et al. used a clinic-based GaAlAs diode laser at 637 nm applied by clinicians, whereas Monea et al. used the OsseoPulse phototherapy device, a low-intensity LED system intended for daily patient use under clinician supervision. Pereira et al. [34] compared two PBM protocols after third molar extractions. Both improved soft tissue healing, edema, and bone density with no difference between protocols [34]. Torkzaban et al. [35] found no significant effect of LLLT on implant stability in low-quality bone, though a slight early healing trend was noted [35]. Table 5 presents the main outcomes of the study.

### 3.6. Characteristics of Light Sources

Table 6 shows the parameters of the lasers used in each study.

## 4. Discussion

### 4.1. Results in the Context of Other Evidence

Across the randomized trials, PBMT demonstrated consistent early benefits for soft tissue healing and inflammatory control, while its effects on hard tissue outcomes were more variable and appeared to depend on treatment protocols [24,27,34]. In periodontal intra-bony defects treated with bioactive glass, adjunctive GaAlAs laser therapy accelerated clinical and radiographic improvements at three months, but these benefits diminished by six months, suggesting that PBMT primarily enhances the early stages of tissue repair [7,12,24]. Similarly, third molar studies using dual-wavelength or diode regimens showed progressive improvements in soft tissue healing and edema reduction, with no clear advantage between intraoral and combined intraoral-extraoral delivery methods, indicating that properly dosed red and near-infrared light can support recovery regardless of application route [27,34]. For implant stability, three well-controlled trials found no significant differences between PBMT and the control groups, despite both groups showing expected time-related increases in implant stability quotient (ISQ), suggesting that baseline surgical stability may overshadow PBMT’s influence in favorable conditions [26,29,35]. One split-mouth trial reported a temporary ISQ advantage at week five with 637 nm PBMT, but this did not correspond to improvements in biochemical markers or implant success rates, indicating that such mechanical gains may have limited clinical relevance [31]. In contrast, histologic evidence from grafted extraction sockets demonstrated that daily PBMT treatments accelerated new bone formation, supporting mechanistic data showing that PBMT-driven mitochondrial signaling can enhance matrix deposition and tissue maturation [7,12,14,32]. Likewise, in adolescents undergoing distraction osteogenesis, PBMT increased bone mineral density and reduced immature bone volume, aligning with findings that red and near-infrared irradiation stimulate osteoblast activity and angiogenesis during bone growth and remodeling [10,11,33]. However, not all trials demonstrated positive outcomes. A high-quality study on periapical lesions found no additional volumetric benefit from a single PBMT session following root canal therapy, highlighting that dosing schedules and total energy delivery are crucial for achieving measurable effects, particularly in complex bone lesion healing [12,30]. Overall, these findings suggest that PBM is a reliable adjunct for improving early soft tissue healing and, in some cases, accelerating bone remodeling, though durable effects on implant osseointegration or bone lesion resolution require optimized multi-session protocols and careful patient selection [24,25,26,27,28,29,30,31,32,33,34,35]. Supporting this, de Barros et al. reported that low-level laser therapy significantly reduced postoperative pain following lower third molar extractions in a systematic review and meta-analysis of 15 randomized clinical trials [36]. Furthermore, Ma et al. demonstrated that PBMT at 635/808 nm enhanced the proliferation and osteogenic differentiation of human umbilical cord mesenchymal stem cells by activating the Akt signaling pathway, increasing P-Akt expression, and boosting ALP activity, mineralized nodule formation, and osteogenesis-related gene expression [37]. Similarly, Li et al. showed that PBM at 650 nm significantly promoted the osteogenic differentiation and mineralization of osteoporotic bone marrow mesenchymal stem cells by upregulating osteogenic markers and activating autophagy, with the inhibition of autophagy reversing these effects [38,39,40,41].

### 4.2. Influence of Clinical and Protocol Heterogeneity

A major challenge in interpreting the effects of PBMT across the included randomized controlled trials is the substantial heterogeneity in clinical indications and treatment protocols. The studies encompassed a wide range of surgical procedures, including third molar extractions, periodontal infra-bony defect treatment, grafted extraction sockets, implant placement in healed and non-healed sites, distraction osteogenesis, and management of chronic periapical lesions. These procedures differ markedly in baseline tissue biology, healing dynamics, and mechanical stability requirements, which inevitably influences responsiveness to adjunctive biostimulatory interventions. In addition to procedural variability, considerable heterogeneity was observed in laser parameters and application protocols. Wavelengths ranged from red (630–660 nm) to near-infrared (808–970 nm), power outputs and energy densities varied widely, and irradiation schedules differed in terms of number of sessions, timing relative to surgery, and mode of delivery (contact vs. non-contact, intraoral vs. combined intraoral–extraoral). Such variability limits a direct comparison between studies and precludes the identification of a single optimal PBMT protocol applicable across all clinical contexts. When outcomes were examined by domain, distinct patterns emerged. The effects of PBMT on early soft tissue healing, postoperative inflammation, and edema reduction were relatively consistent across studies, regardless of surgical indication or specific laser parameters. This suggests that PBMT exerts a reproducible biological effect on early inflammatory modulation and epithelial repair. In contrast, bone-related outcomes, including bone regeneration, mineral density, and implant stability, demonstrated greater variability and appeared to be more dependent on surgical context, baseline bone quality, and dosing regimens. For example, beneficial effects were more frequently observed in grafted extraction sockets and distraction osteogenesis models, whereas studies evaluating implant stability in sites with high primary stability often reported no additional benefit. Long-term outcomes, such as chronic periapical lesion healing and sustained implant osseointegration, showed the greatest inconsistency, likely reflecting both biological complexity and insufficient standardization of treatment protocols and follow-up durations. These findings indicate that PBMT should not be viewed as a uniform intervention with identical effects across all postoperative scenarios, but rather as a context-dependent adjunct whose efficacy is influenced by procedure type, tissue depth, and dosing strategy. Overall, this heterogeneity limits the comparability of the included trials and constrains the strength of generalized clinical recommendations. At the same time, it underscores the need for future randomized controlled trials using standardized reporting of surgical protocols, baseline bone characteristics, and laser parameters. Establishing consensus-based PBMT protocols tailored to specific surgical indications will be essential to improve reproducibility, enable the meaningful synthesis of evidence, and clarify the clinical contexts in which PBMT provides the greatest benefit.

### 4.3. Limitations of the Evidence

The current evidence base evaluating laser-based biostimulation in postoperative osteogenesis presents several important limitations. A major challenge is the considerable heterogeneity across studies, including differences in surgical procedures, anatomical sites of bone healing, laser types, wavelengths, power outputs, energy densities, treatment durations, and application protocols. This variability makes it difficult to identify optimal treatment parameters and limits the generalizability of the findings. Many trials included in this review also had relatively small sample sizes, reducing statistical power and increasing the risk of type II error. Follow-up periods were often short, restricting conclusions regarding the long-term durability of treatment effects and the potential for late complications or relapse. In addition, outcome measures varied widely, with some studies relying on radiographic assessment, others using histological analysis, and others reporting only clinical indicators of healing, making direct comparisons challenging. Inconsistent or incomplete reporting of patient characteristics, such as age, systemic health status, smoking habits, or use of adjunctive therapies, further complicates the interpretation of results and may mask potential confounding effects. Finally, there is a limited number of high-quality randomized controlled trials in this area, and many existing studies do not fully adhere to established reporting standards. Addressing these limitations through large-scale, multicenter, methodologically rigorous RCTs with standardized protocols and extended follow-up will be essential to strengthen the evidence supporting the clinical use of laser-based biostimulation in postoperative bone healing.

### 4.4. Limitations of the Review Process

Several limitations should be acknowledged regarding the review process undertaken in this systematic evaluation of laser-based biostimulation in postoperative osteogenesis. First, although a comprehensive and structured search strategy was implemented, only English-language publications were considered, which may have introduced language bias and excluded potentially relevant studies in other languages. Second, the decision to exclude grey literature, conference proceedings, and unpublished data may have increased the risk of publication bias, as studies with negative or inconclusive results are less likely to be published in peer-reviewed journals. Third, variations in study design, surgical procedures, laser device specifications, irradiation protocols, and outcome measurement methods limited the feasibility of performing a quantitative meta-analysis and made direct comparisons between trials challenging. Fourth, the reliance on reported data meant that inconsistencies or incomplete descriptions of treatment parameters, follow-up durations, and patient characteristics could not be clarified, increasing the possibility of reporting bias. Finally, although the review process incorporated independent screening and data extraction by multiple reviewers to reduce selection bias, the subjectivity inherent in eligibility assessment and interpretation of results cannot be entirely eliminated. These limitations highlight the need for more standardized protocols, consistent reporting of methodological details, and the inclusion of a broader evidence base in future systematic reviews on this topic.

### 4.5. Implications for Practice, Policy, and Future Research

PBM shows promise as a safe, non-invasive adjunctive therapy to enhance early postoperative soft tissue healing, control inflammation, and, in selected cases, accelerate bone remodeling. Evidence from this review indicates that PBM is most effective in the early stages of recovery, with benefits observed in grafted extraction sockets, distraction osteogenesis, and third molar surgeries, while its impact on long-term outcomes such as implant stability and resolution of chronic periapical lesions remains inconsistent. Clinically, PBM should not replace standard surgical protocols but be used to complement them, especially in patients with compromised bone biology or systemic risk factors such as diabetes, smoking, or osteoporosis. Optimal application requires careful control of dosing parameters, with red light (630–660 nm) recommended for soft tissue and near-infrared light (808–970 nm) for deeper bone, per-point energies typically ranging from 4–10 J for soft tissue and 8–40 J for bone, delivered immediately after surgery and repeated over the first 1–3 weeks when regenerative activity is highest. Standardized documentation should include wavelength, power output, beam size, energy per point, exposure time, and session frequency, with strict adherence to eye protection and contraindication screening.

At the policy level, institutions should implement clear PBM protocols, regular device calibration, and operator training, while regulators and journals should mandate transparent reporting of laser parameters and treatment protocols following CONSORT and TIDieR guidelines to improve reproducibility and reduce heterogeneity. From a health system perspective, coverage decisions should focus on indications with the strongest evidence of benefit, such as enhanced early bone formation and reduced postoperative morbidity, and tie reimbursement to standardized outcomes and quality control. Future research must address current gaps by conducting large, multicenter randomized controlled trials with standardized protocols and longer follow-up to assess sustained bone regeneration, using consistent outcome measures such as pain, edema, soft tissue healing indices, radiographic bone density, implant stability quotient (ISQ) trajectories, and adverse events. Studies should also explore dose–response relationships, optimal session timing, and patient-related factors influencing PBM efficacy, as well as direct comparisons with other regenerative modalities like platelet-rich fibrin. Real-world registries and independent calibration studies are needed to ensure device accuracy, identify safety signals, and support implementation. In summary, PBM currently offers reliable improvements in early healing and may accelerate bone regeneration in specific surgical scenarios, but widespread clinical adoption requires standardized dosing guidelines and high-quality evidence defining the patient populations and surgical contexts where it delivers meaningful, long-term benefits.

An additional consideration is the potential role of home-use laser or LED devices, which allow patients to perform photobiomodulation therapy independently under professional guidance. Such systems could enhance treatment adherence and enable more frequent, low-dose applications that may sustain biostimulatory effects between clinical visits. Integrating home-based PBM into postoperative protocols could therefore improve the convenience, consistency, and overall healing outcomes, although device safety, calibration, and patient training remain essential to ensure effective and standardized use.

## 5. Conclusions

This systematic review of randomized controlled trials evaluated the effects of laser-based biostimulation on postoperative osteogenesis. The findings indicate that PBM consistently improves early postoperative soft tissue healing and reduces inflammation, with some evidence supporting its ability to accelerate early bone remodeling in specific clinical scenarios, such as grafted extraction sockets and distraction osteogenesis. However, its impact on long-term outcomes, including implant stability, complete resolution of chronic periapical lesions, and sustained bone regeneration, remains inconsistent and highly dependent on treatment parameters such as wavelength, energy dose, and frequency of application. The considerable heterogeneity among studies highlights the need for standardized protocols to guide clinical practice and ensure reproducibility. While PBM appears to be a safe and low-burden adjunct to surgical care, further high-quality, multicenter RCTs with extended follow-up and standardized outcome measures are required to determine the optimal dosing regimens, identify the patient populations most likely to benefit, and clarify its role in routine postoperative bone management. Until such evidence is available, PBM should be considered as a supportive therapy rather than a stand-alone intervention for enhancing postoperative osteogenesis.

## Figures and Tables

**Table 1:** Search syntax used in the study.

Source	Search Terms	Filters	Number of Results
PubMed	(low level laser therapy OR LLLT OR laser biostimulation OR photobiomodulation OR photobiomodulation therapy OR light emitting diode OR LED therapy OR diode laser OR erbium laser OR neodymium laser OR Nd:YAG OR CO2 laser OR GaAlAs OR gallium aluminum arsenide OR holmium laser OR infrared laser OR therapeutic laser) AND (postoperative OR post operative OR postsurgical OR after surgery) AND (osteogenesis OR bone regeneration OR bone healing OR bone formation OR osseointegration)	RCT	14
Embase	(low level laser therapy OR lllt OR laser biostimulation OR photobiomodulat* OR light emitting diode OR led therapy OR diode laser OR erbium laser OR neodymium laser OR nd:yag OR co2 laser OR gaalas OR gallium aluminum arsenide OR holmium laser OR infrared) AND (postoperative OR post operative OR postsurgical OR after surgery) AND (osteogenesis OR bone regeneration OR bone healing OR bone formation OR osseointegration) AND (random* OR placebo OR trial OR controlled clinical trial)	RCT	20
Scopus	TITLE-ABS-KEY(low level laser therapy OR LLLT OR laser biostimulation OR photobiomodulat* OR light emitting diode OR LED therapy OR diode laser OR erbium laser OR neodymium laser OR Nd:YAG OR CO2 laser OR GaAlAs OR infrared laser) AND TITLE-ABS-KEY(postoperative OR post operative OR postsurgical OR after surgery) AND TITLE-ABS-KEY(osteogenesis OR bone regeneration OR bone healing OR bone formation OR osseointegration) AND TITLE-ABS-KEY(random* OR placebo OR trial)	Article	19
Cochrane Library	(low level laser therapy OR LLLT OR laser biostimulation OR photobiomodulat* OR light emitting diode OR LED therapy OR diode laser OR erbium laser OR neodymium laser OR Nd:YAG OR CO2 laser OR GaAlAs OR infrared laser) AND (postoperative OR post operative OR postsurgical OR after surgery) AND (osteogenesis OR bone regeneration OR bone healing OR bone formation OR osseointegration) AND (random* OR placebo OR trial)	—	40

**Table 2:** Quality assessment for the included studies.

Study	D1	D2	D3	D4	D5	Overall RoB
AboElsaad et al., 2009 [24]	* 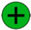 *	* 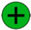 *	* 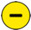 *	* 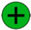 *	* 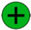 *	* 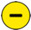 *
Akkaya et al., 2023 [25]	* 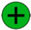 *	* 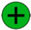 *	* 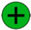 *	* 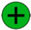 *	* 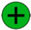 *	* 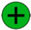 *
Bozkaya et al., 2020 [26]	* 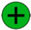 *	* 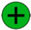 *	* 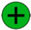 *	* 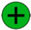 *	* 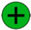 *	* 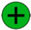 *
Camolesi et al., 2022 [27]	* 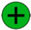 *	* 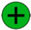 *	* 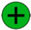 *	* 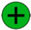 *	* 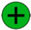 *	* 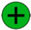 *
Demirok et al., 2024 [28]	* 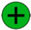 *	* 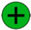 *	* 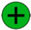 *	* 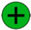 *	* 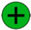 *	* 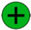 *
García-Morales et al., 2011 [29]	* 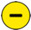 *	* 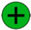 *	* 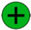 *	* 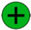 *	* 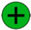 *	* 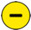 *
Ismail et al., 2025 [30]	* 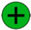 *	* 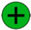 *	* 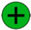 *	* 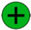 *	* 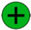 *	* 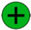 *
Mandić et al., 2015 [31]	* 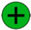 *	* 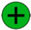 *	* 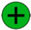 *	* 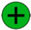 *	* 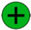 *	* 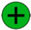 *
Monea et al., 2015 [32]	* 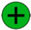 *	* 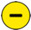 *	* 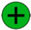 *	* 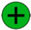 *	* 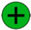 *	* 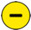 *
Özkan et al., 2015 [33]	* 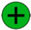 *	* 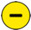 *	* 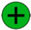 *	* 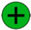 *	* 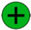 *	* 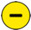 *
Pereira et al., 2024 [34]	* 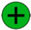 *	* 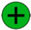 *	* 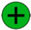 *	* 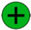 *	* 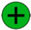 *	* 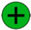 *
Torkzaban et al., 2017 [35]	* 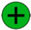 *	* 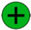 *	* 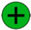 *	* 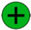 *	* 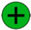 *	* 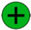 *

D1—Randomization, D2—Deviations from interventions, D3—Missing outcome data, D4—Outcome measurement, D5—Selective reporting, 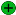
—low, 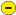
—some concerns.

**Table 3:** GRADE quality of evidence.

Outcome	No. of Studies (Participants)	Study Design	Risk of Bias	Inconsistency	Indirectness	Imprecision	Publication Bias	Overall Certainty of Evidence
Early soft tissue healing	6 RCTs (~180 participants)	Randomized controlled trials	Not serious	Not serious	Not serious	Not serious	Undetected	High ⊕⊕⊕⊕
Postoperative pain and edema	5 RCTs (~170 participants)	Randomized controlled trials	Not serious	Not serious	Not serious	Not serious	Undetected	High ⊕⊕⊕⊕
Bone regeneration/bone density	5 RCTs (~140 participants)	Randomized controlled trials	Not serious	Serious	Not serious	Not serious	Undetected	Moderate ⊕⊕⊕◯
Implant stability (ISQ)	5 RCTs (~200 implants)	Randomized controlled trials	Not serious	Serious	Not serious	Not serious	Undetected	Moderate ⊕⊕⊕◯
Healing of chronic periapical lesions	1 RCT (60 participants)	Randomized controlled trial	Not serious	Not applicable	Not serious	Not serious	Undetected	Moderate ⊕⊕⊕◯
Adverse events/safety	7 RCTs (~250 participants)	Randomized controlled trials	Not serious	Not serious	Not serious	Not serious	Undetected	High ⊕⊕⊕⊕

RCT—Randomized controlled trial.

**Table 4:** Main characteristics of the included studies.

Study	Country	Aim
AboElsaad et al., 2009 [24]	Egypt & UK	To investigate clinically and radiologically the influence of low-level GaAlAs on the healing of infra-bony defects filled with bioactive glass.
Akkaya et al., 2023 [25]	Turkey	To evaluate and compare the effects of PRF, diode laser, and their combination on gingival blood perfusion and early bone healing after tooth extraction.
Bozkaya et al., 2020 [26]	Turkey	To assess whether PBMT improves implant stability and affects the microbiota around dental implants during the early stage of osseointegration.
Camolesi et al., 2022 [27]	Spain	To evaluate the effect of diode laser PBMT on post-surgical healing, inflammation, and implant stability.
Demirok et al., 2024 [28]	Turkey	To compare L-PRF and PBMT in terms of pain, soft tissue, and bone healing in tooth extraction sockets.
García-Morales et al., 2011 [29]	Brazil	To investigate whether LLLT enhances the stability of titanium implants during osseointegration using RFA.
Ismail et al., 2025 [30]	Egypt	To evaluate the healing of chronic periapical bone lesions after root canal therapy with or without diode laser application, using CBCT to measure changes in lesion volume.
Mandić et al., 2015 [31]	Serbia	To investigate the influence of postoperative LLLT on the osseointegration and early success of self-tapping implants placed into low-density bone in the posterior maxilla.
Monea et al., 2015 [32]	Romania	To determine whether LLLT can reduce the time between extraction/socket graft and implant placement by evaluating histological changes in grafted sockets treated with LLLT.
Özkan et al., 2015 [33]	Turkey	To evaluate the effect of LLLT on bone mineral density and volume during mandibular midline distraction osteogenesis using high-resolution CT and stereology.
Pereira et al., 2024 [34]	Brazil	To compare the effects of different dual-wavelength photobiomodulation protocols on soft tissue and bone healing after third molar extractions.
Torkzaban et al., 2017 [35]	Iran	To evaluate the efficacy of LLLT using a 940 nm diode laser on the stability of dental implants through a randomized controlled clinical trial.

LLLT—Low-Level Laser Therapy, PBMT—Photobiomodulation Therapy, GaAlAs—Gallium–Aluminum–Arsenide Laser, PRF—Platelet-Rich Fibrin, L-PRF—Leukocyte and Platelet-Rich Fibrin, RFA—Resonance Frequency Analysis, CBCT—Cone Beam Computed Tomography, CT—Computed Tomography.

**Table 5:** Summary of principal results and study details.

Author (Year)	Study Groups	Main Outcomes
AboElsaad et al., 2009 [24]	20 patients, split-mouth infrabony defects. Test: bioactive glass + GaAlAs laser. Control: bioactive glass alone.	Significant improvement in PPD, CAL, and bone fill at 3 months in laser group; no difference at 6 months.
Akkaya et al., 2023 [25]	40 premolar sockets, 4 groups: control, PRF, diode laser, PRF + laser.	PRF + laser showed greater early bone formation vs. control; perfusion changes similar across groups.
Bozkaya et al., 2020 [26]	22 patients, 93 implants. PBMT vs. sham laser.	ISQ increased in both groups; no significant effect of PBMT on implant stability or microbiology.
Camolesi et al., 2022 [27]	13 patients, 40 implants. Dual-wavelength PBMT vs. sham.	PBMT improved early soft tissue healing and reduced inflammation; implant stability unchanged.
Demirok et al., 2024 [28]	34 patients, split-mouth third molars. L-PRF vs. PBMT.	No significant differences in pain, healing index, or bone density; both methods supported healing.
García-Morales et al., 2011 [29]	8 patients, 30 implants. GaAlAs LLLT vs. placebo.	No significant differences in implant stability at any time point.
Ismail et al., 2025 [30]	60 patients with periapical lesions. Diode PBMT vs. mock laser.	Lesion volume decreased in both groups; PBMT provided no additional benefit.
Mandić et al., 2015 [31]	12 patients, 44 implants. Postoperative LLLT vs. no laser.	Higher stability at week 5 with LLLT; no effect on ALP or overall osseointegration.
Monea et al., 2015 [32]	30 grafted extraction sockets. LLLT vs. control.	Reduced healing time with LLLT; earlier readiness for implant placement.
Özkan et al., 2015 [33]	9 adolescents undergoing mandibular distraction. LLLT vs. control.	Higher bone mineral density and faster bone maturation with LLLT.
Pereira et al., 2024 [34]	17 patients, split-mouth third molars. Intraoral vs. intra+extraoral PBMT.	Both protocols improved healing and bone density; no differences between approaches.
Torkzaban et al., 2017 [35]	19 patients, 80 implants in D3–D4 bone. LLLT vs. sham.	Implant stability increased over time in both groups; no significant laser effect.

ALP—Alkaline Phosphatase, GaAlAs—Gallium-Aluminum-Arsenide, ISQ—Implant Stability Quotient, L-PRF—Leukocyte and Platelet-Rich Fibrin, LLLT—Low-Level Laser Therapy, PBMT—Photobiomodulation Therapy, PRF—Platelet-Rich Fibrin.

**Table 6:** Characteristics of light source used.

Study (First Author, Year)	Laser Type & Model	Wavelength	Power/Energy/Mode	Number of Applications	Delivery & Settings
AboElsaad et al., 2009 [24]	GaAlAs Diode Laser, Velopex (MeDivance Instruments Ltd., London, UK)	830 nm	40 mW, CW; fluence: 4 J/cm^2^; total energy density: 16 J/cm^2^	Not specified	400 μm optical fibers, 1 cm away from tissue, spot size 3 mm, exposure time 60 s per application
Akkaya et al., 2023 [25]	GaAlAs Diode Laser, Epic X (Biolase, USA)	940 nm	0.2 W; energy density 10 J/cm^2^; CW; 25 s per point	3	Irradiation on occlusal and buccal mid-socket areas, performed immediately post-op and on days 1, 3, and 7
Bozkaya et al., 2020 [26]	GaAlAs Diode Laser, BTL 4110 Laser Professional (BTL Industries, UK)	830 nm	Power: 300 mW; average output: 126 mW; energy per point: 0.3 J; total energy: 6 J; energy density: 135 J/cm^2^	Not specified	Non-contact technique, 0.5–1 cm distance, 20 points around implant (vestibular, lingual, distal, mesial); 3 s per point; continuous wave; three times/week for 2 weeks
Camolesi et al., 2022 [27]	Diode Laser, Laser Duo (MMO-São Carlos, Brazil)	Dual wavelengths: 630 nm (soft tissue) and 808 nm (bone tissue)	Output power: 100 mW; dose: 0.1 J/s; continuous mode	2	Handpiece with 3 mm^2^ output tip; applied immediately post-surgery and at day 7
Demirok et al., 2024 [28]	InGaAsP Diode Laser, Biolase Epic X (Biolase Technology Inc., Irvine, CA, USA)	940 nm	Power density: 0.5 W/cm^2^; Continuous mode; Total energy per application: 30 J per point (60 J total/session)	7	Intraoral non-contact, buccal + occlusal; 2 points irradiated for 60 s each; 7 sessions on days 2, 4, 7, 11, 14, 18, and 21
García-Morales et al., 2011 [29]	GaAlAs Diode Laser, Thera Lase (DMC, São Carlos—SP, Brazil)	830 nm	Power: 86 ± 2 mW; Energy per point: 0.25 J; Energy density: 92.1 J/cm^2^; 3 s per point; Continuous emission	7	Punctual contact technique, 20 points per implant (9 vestibular, 9 lingual, 1 distal, 1 mesial); 7 irradiation sessions every 48 h for 14 days
Ismail et al., 2025 [30]	Diode Laser, LITEMEDICS (Serial Number: 148, SwvM.150VS108VT.100)	970 nm	Power: 0.5 W; Energy per point: 30 J; Total energy: 60 J; CW, 10 Hz	1	Contact mode, no movement; two points at the level of the targeted root apex (buccal and lingual); single session immediately after root canal treatment
Mandić et al., 2015 [31]	GaAlAs Diode Laser, Medicolaser 637 (Technoline, Belgrade, Serbia)	637 nm	40 mW, CW	8	Irradiation intraorally, orthoradially to implant axis; immediately post-op and daily for 7 days; total dose per treatment: 6.26 J/cm^2^ per implant
Monea et al., 2015 [32]	OsseoPulse phototherapy device	Not reported	Intensity: 20 mW/cm^2^; 20 min per session	21	Delivered daily for 21 consecutive days to extraction sockets grafted with allograft
Özkan et al., 2015 [33]	GaAlAs Diode Laser	830 nm	40 mW; 8.4 J/cm^2^ per spot	8	Applied from 2 midline mandibular points; 8 treatment sessions at 48 h intervals
Pereira et al., 2024 [34]	GaAlA Laser, Therapy EC (DMC Equipamentos, São Carlos, Brazil)	Dual wavelengths: 660 nm (red) and 808 nm (infrared)	100 mW, CW; total energy: 32 J per session, 96 J total across 3 sessions	3	PBMT-I: Dual wavelengths applied intraorally. PBMT-IE: 660 nm intraorally, 808 nm transcutaneously at four masseter points per quadrant.
Torkzaban et al., 2017 [35]	Diode Laser, epic10 (BIOLASE, Irvine, CA, USA)	940 nm	100 mW output power; CW; 4 J each side per session (8 J total per session)	7	Deep tissue handpiece, 6 mm spot size; applied to buccal and palatal sides for 40 s each; 7 sessions on days 2, 4, 6, 8, 10, and 12 post-op

CW—Continuous Wave, InGaAsP—Indium Gallium Arsenide Phosphide, PBMT-I—Photobiomodulation Therapy Intraoral, PBMT-IE—Photobiomodulation Therapy Intraoral and Extraoral.

## Data Availability

Not applicable.

## Supplementary Materials

The following supporting information can be downloaded at: https://www.mdpi.com/article/10.3390/jcm15020613/s1, PRISMA 2020 Checklist.
